# Letter on “Sigh maneuver to enhance assessment of fluid responsiveness during pressure support ventilation”

**DOI:** 10.1186/s13054-019-2457-y

**Published:** 2019-05-24

**Authors:** Khaoula Meddeb, Mohamed Boussarsar

**Affiliations:** 1 Medical Intensive Care Unit, Farhat Hached University Hospital, 4000 Sousse, Tunisia; 2 Research Laboratory No. LR12SP09, Heart Failure, Faculty of Medicine of Sousse, University of Sousse, Sousse, 4000 Tunisia

For the past years, sigh maneuver was mainly studied as one of many techniques to lung recruitment in acute respiratory distress syndrome [1]. Hemodynamic effects observed during these techniques were mainly related to the increase of intrathoracic pressures [2].

We read, with great attentiveness, the recent article by Messina et al. [3], a prospective bi-centric interventional study on sigh maneuver as a tool to assess fluid responsiveness during pressure support ventilation (PSV). The study was conducted among 40 critically ill patients with a stable ventilatory PSV pattern and requiring volume expansion. Variations in systolic arterial pressure (SAP), pulse pressure (PP), and stroke volume index (SVI) were assessed consequent to random application of 4-s sighs at three different inspiratory pressures; 15 cmH_2_O, 25 cmH_2_O, and 35 cmH_2_O. The authors concluded that the analysis of the slope for SAP after the application of three successive sighs and the nadir of PP after Sigh35, reliably predict fluid responsiveness [3].

Perel et al. published a previous study suggesting that a standardized ventilatory maneuver consisting of a series of successive incremental pressure-controlled breaths may be useful in guiding fluid therapy in ventilated patients [4]. Certainly, Messina et al. displayed a meticulous approach to applying the presented method concluding to compelling results; however, a few questions could be raised.

First, the authors’ choice to use incremental increase in pressure support as a way to implement the sigh maneuver may be reasonable for patients under pressure support ventilation; nonetheless, this method has some limits. Setting pressure requires flow and volume monitoring which can be challenging. In fact, depending on underlying respiratory mechanics, flow varies greatly to maintain identical high pressure leading to volume fluctuation. For instance, in patients with resistive respiratory mechanics, setting pressure at 35 cmH_2_O, could possibly fail to reach a significant variation in volume capable of inducing right ventricle preload and afterload changes. It could be informative if the author shared the volume ranges generated by the different set pressures, especially at 35 cmH_2_O, and what respiratory mechanics did the study population present at the time.

Second, the study population seemed to be overly selected, patients were under low pressure support levels with no hypoxemia which leads to doubts regarding this method generalizability, especially for patients with impaired respiratory mechanics.

Although the results displayed by Messina et al. [3] seem promising, further investigations are needed especially on less selected study population.

## Abbreviations

PP: Pulse pressure
PSV: Pressure support ventilation
SAP: Systolic arterial pressure
SVI: Stroke volume index
