# Supplementary material for: Letter on “Sigh maneuver to enhance assessment of fluid responsiveness during pressure support ventilation”
Source: Crit Care. 2019 May 24;23:188. doi: 10.1186/s13054-019-2457-y (PMC6533664; doi:10.1186/s13054-019-2457-y)
Supplement: Related texts

# Reply: Authors’ response

**Authors:** Antonio Messina, Davide Colombo, Maurizio Cecconi, Paolo Navalesi

Thank you for giving us the chance to reply to the valuable comments raised by Meddeb regarding our article [3]. The number of ventilated intensive care unit (ICU) patients, with spontaneous breathing activity, is overall increasing [5]. In this subgroup of patients, the dynamic preload indices are often not applicable.

To the best of our knowledge, this is the first hemodynamic test specifically designed for ICU patients undergoing pressure support ventilation (PSV). As pointed out by Meddeb and Boussarsar, the slope analysis of systolic pressure changes after the delivery of consecutive and incremental pressure-controlled inspiratory breaths has been previously tested in postsurgical patients without spontaneous breathing activity [4, 6]. Our results show that this approach could be useful also during PSV, being however, far from bedside application.

The increase in intrathoracic pressure occurring during the sigh increases lung volume to an extent that varies depending on the extra pressure applied to the airway and on the mechanical properties of the respiratory system. The higher the increase in Paw and the lower the respiratory system impedance, the larger the volume generated during the sigh. We did not measure these changes, assuming that, in a homogenous population, irrespective to the absolute volume obtained by the sigh, the final hemodynamic effect would be comparable. For sure, in some patients the application of standard sigh maneuver of 35 cmH_2_0 for 4 s could trigger a drop of pulse pressure and stroke volume insufficient (leading to false negative results) or excessive (leading to false positive results) to assess fluid responsiveness. In this “proof of concept” study, we set the highest level of inspiratory support, according to the harmless maneuver already used by Patroniti et al. in ICU patients with early acute respiratory distress syndrome [7]. Moreover, as pointed out in their Letter and as stated in our paper, some inclusion and exclusion criteria of our study would limit the external validity of the results. This choice was made to reduce the risk of bias due to the number of potential interferences between a beat-to-beat analysis and the variable respiratory pattern of ICU patients undergoing partial ventilatory support. We hope that our results would encourage further investigations in this group of patients.
